# Hemorrhagic Lumbar Synovial Cyst: Rare Mimic for Mass Lesion Causing Acute Neurological Deficit

**DOI:** 10.1227/neuprac.0000000000000246

**Published:** 2026-06-02

**Authors:** Andrea C. Zoana, Jose Solis, Chandan G. Reddy

**Affiliations:** 1 Neuroscience Institute, AdventHealth, Orlando, Florida, USA;; 2 Department of Medicine, Universidad de Ciencias Médicas, San José, Costa Rica;; 3 Department of Neurosurgery, AdventHealth Celebration, Celebration, Florida, USA;; 4 Orlando Neurosurgery, Orlando, Florida, USA

**Keywords:** Case report, Hemorrhagic synovial cyst, Lumbar synovial cyst, Hemorrhagic cyst

## Abstract

**BACKGROUND AND IMPORTANCE:**

Spinal epidural masses are often associated with neoplastic, infectious, or inflammatory etiologies. However, hemorrhagic epidural lesions can mimic these pathologies on imaging, presenting a serious diagnostic challenge.

**CLINICAL PRESENTATION:**

This case report presents 2 patients with distinct hemorrhagic epidural masses causing acute bilateral lower extremity weakness. First case is a 63-year-old female presenting with progressive weakness following a cruise, found to have an L3-L5 enhancing epidural mass mimicking an abscess or tumor. Second case is a 55-year-old male with new onset of radiculopathy and weakness, found to have an L4-L5 mass initially suspected to be an intradural tumor. In both instances, advanced imaging was nondiagnostic, and final pathology confirmed benign, hemorrhagic processes: a hemorrhagic synovial cyst in Case 1 and an extradural synovial cyst in Case 2.

**CONCLUSION:**

These cases highlight the importance of including hemorrhagic synovial cysts in the differential diagnosis of acute spinal compression, regardless of the patient's oncologic history or atypical presentation. Our findings underscore the importance of timely surgical decompression and definitive histopathological analysis for accurate diagnosis and optimal neurological recovery.

Spinal epidural masses are often associated with neoplastic, infectious, or inflammatory etiologies. Hemorrhagic synovial cysts, although rare, are a notable diagnostic consideration, particularly in patients presenting with acute or progressive neurological decline. These cysts, typically adjacent to the facet joints, most frequently affect the lumbar spine, especially the L4-L5 level, being strongly linked to degenerative joint changes and segmental instability.^1^

Although synovial cysts are often asymptomatic or cause slowly progressive radicular symptoms, hemorrhage within a cyst can lead to acute spinal cord or nerve roots compression. Such presentations can mimic tumors or epidural abscesses on imaging, complicating the diagnostic process.^2,3^ In fact, when hemorrhagic, synovial cysts may show contrast enhancement and mass effect on MRI, resembling neoplastic or infectious lesions.^4^

Because imaging alone cannot reliably distinguish these entities, definitive diagnosis often requires surgical resection and histopathological analysis.^1-4^ Both our cases illustrate this challenge. The first case mimicked an epidural lesion, initially suspected to be infectious or malignant, and ultimately diagnosed as organizing hemorrhage. Our second case was originally considered to be an intradural lesion based on imaging, oncologic history, and even initial intraoperative findings. These underscore the need to include hemorrhagic synovial cysts in the differential diagnosis of enhancing epidural masses, particularly when infectious workup is negative and neurological symptoms progress rapidly.

## CLINICAL PRESENTATION

### Case 1

#### Case Description

The patient is a 63-year-old female with a history of chronic sciatica, obesity, hypertension, and no known prior diagnosis of malignancy or immunosuppression. She presented with a 4-week history of lower back pain and 1-week history of bilateral lower extremity weakness, which occurred suddenly while on a cruise. She denied trauma but sustained a fall around the time of onset of weakness.

On neurological examination, she exhibited 4/5 strength in both lower extremities, absent deep tendon reflexes, and intact sensation, without saddle anesthesia, urinary retention, or incontinence.

#### Diagnostic Assessment

**FIGURE 1.**
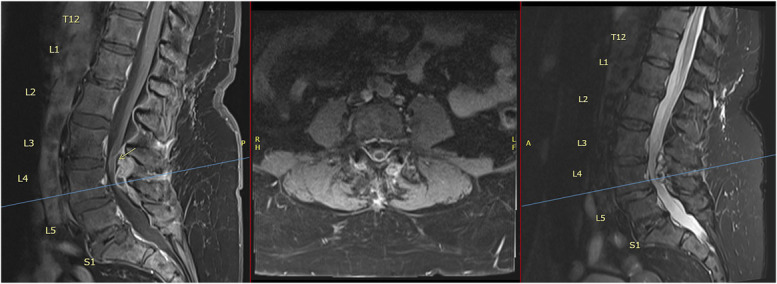
Preoperative MRI—Case 1. The sagittal (left) and axial (center) T1-weighted MRI with contrast and sagittal T2 (right) demonstrate the presence of a posterior epidural lesion at the L3-4 level (yellow arrow) and L4-5 levels with significant compression of the thecal sac and moderate to severe spinal canal narrowing. The axial T1-weighted image with contrast (center) shows a facet joint-associated posterolateral epidural lesion corresponding to the synovial cyst, with peripheral contrast enhancement.

Initial MRI of the lumbar spine revealed multilevel degenerative changes with severe canal stenosis at the L3-4 and L4-5 levels (Figure 1). A ventral epidural lesion was identified, which exhibited contrast enhancement and caused significant mass effect on the thecal sac.

Differential diagnosis included epidural abscess, hematoma, and neoplastic lesion. However, laboratory workup showed normal WBC count and inflammatory markers, with negative blood cultures, arguing against an active infectious process. Following unremarkable infectious disease and oncology workup, organized hematoma rose to the top of our differential diagnosis.

#### Therapeutic Intervention

The patient underwent decompressive L3-5 laminectomy with resection of the ventral epidural lesion. Intraoperative findings revealed a dark, discolored epidural mass adherent to the dura, suggestive of chronic hematoma. The mass was carefully dissected from the dura and sent for pathologic analysis, which confirmed an organizing hematoma with no evidence of a neoplasm or infection.

#### Follow-up and Outcomes

**FIGURE 2.**
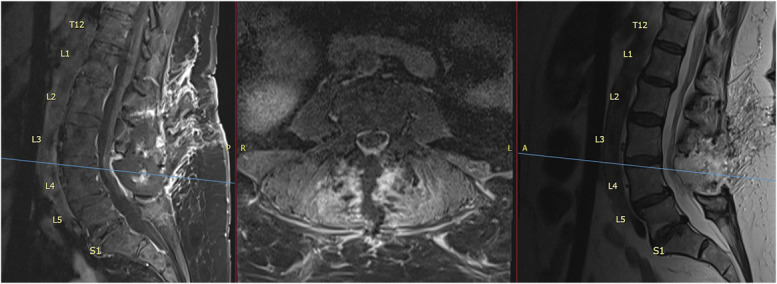
Postoperative MRI—Case 1. The sagittal T1 weighted MRI with contrast (left), axial T1-weighted with contrast (center), and sagittal T2 (right) images demonstrate complete resection of the posterior epidural lesion with restoration of the thecal sac at L4-5 and confirm the absence of the previously noted facet-associated epidural mass.

Postoperatively, the patient began physical rehabilitation, demonstrating gradual recovery of motor function, with rapid strength recovery in the first week. At the 2-month follow-up, she reported normalization of neurologic strength, with bilateral strength improvement. She was ambulating short distances without a walker, although still requiring assistance for longer distances. Her follow-up MRI confirmed complete resection of the epidural lesion (Figure 2). She denied residual pain or neurological deficits and was cleared to continue physical therapy.

### Case 2

#### Case Description

The patient is a 55-year-old male with history of obesity, prediabetes, hairy cell leukemia, and prior L2 kyphoplasty and laminectomy for a compression fracture. He presented after sustaining a lower back injury from a fall 2 months prior, followed by the onset of left toe numbness with progression to bilateral pulling-like sensation in his glutes and hamstrings. When standing or walking, he also experienced a burning sensation in the anterior part of his foot, extending to his toes.

Neurological examination showed 5/5 motor strength in all extremities, with absent sensation in the great toe. He denied saddle anesthesia and bowel or bladder dysfunction.

#### Diagnostic Assessment

**FIGURE 3.**
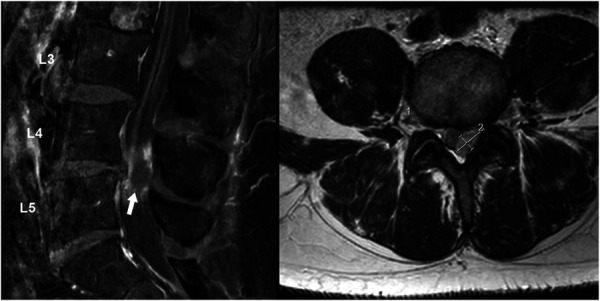
Preoperative MRI—Case 2. The sagittal T1-weighted (left) and axial T2-weighted (right) images reveal a lobulated, heterogeneously enhancing lesion of the left L4-5 cauda equina nerve roots (white arrow, left), resulting in severe canal stenosis and compression of the L5 nerve roots.

MRI of the lumbar spine revealed a lobulated heterogeneously enhancing lesion within the cauda equina nerve roots at the left L4-L5 level, concerning for an underlying neoplasm and causing severe spinal canal stenosis (Figure 3). Given the patient's history of malignancy and the mass effect noted on imaging, the lesion was initially assessed as a presumptive intradural extramedullary spinal tumor.

#### Therapeutic Intervention

**FIGURE 4.**
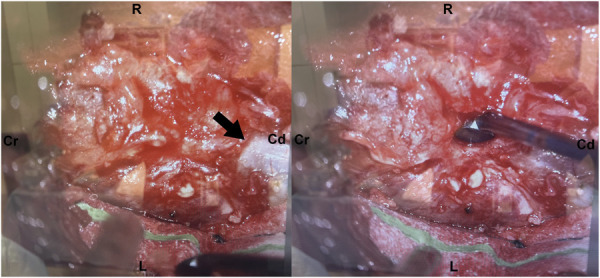
Intraoperative image—Case 2. The left intraoperative image illustrates orientation within the surgical field (Cr, cranial; Cd, caudal; R, right; L, left) and demonstrates the presence of a hemorrhagic synovial cyst exposed at the L4-5 level. The left image shows the adherence of the interface of the cyst with the dura mater (black arrow). The right image shows the adherent quality of the cyst despite the use of a Rhoton microdissector.

The patient underwent L4-L5 laminectomy with the initial plan for intradural tumor resection. Intraoperatively, a left-sided mass was identified, adherent to the dura (Figure 4). After opening the dura for exploration, no intradural tumor was visible, confirming the mass was entirely extradural. Microdissection was used to free the mass from the dura and achieve complete decompression of the thecal sac and exiting nerve roots. The intraoperative frozen section was suggestive of a synovial cyst, ultimately confirmed by the final pathology report.

#### Follow-up and Outcomes

**FIGURE 5.**
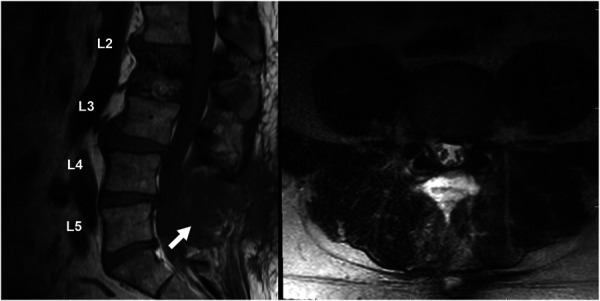
Postoperative MRI—Case 2. The sagittal T1-weighted (left) and axial T2-weighted (right) images demonstrate complete resolution of the posterior epidural lesion at L4-5 (white arrow, left) and confirm the decompression of the spinal canal with no residual mass.

The patient tolerated the surgery well and was mobilized with physical and occupational therapy. He reported significant subjective improvement in left foot numbness and sensitivity. At 1-month follow-up, his postoperative MRI confirmed complete lesion resection (Figure 5). Despite mild residual numbness in his left foot, the patient remained well, being able to ambulate with full strength and no pain.

Given the retrospective nature of the report and complete deidentification of patient information, additional written consent for publication was not required. To ensure privacy compliance, the report was properly deidentified using the Health Insurance Portability and Accountability Act Safe Harbor method and reviewed by our institution's Privacy and Risk Management offices. In accordance with institutional policy and federal regulations, Institutional Review Board or ethics committee approval was not required.

## DISCUSSION

Most synovial cysts develop slowly, causing radicular pain or neurogenic claudication as a result of gradual spinal canal narrowing. However, hemorrhagic conversion can lead to sudden neurologic deterioration, including cauda equina syndrome or acute radiculopathy.^1,4,5^

Our cases highlight the broad clinical spectrum of this acute pathology. The first case demonstrated an acute onset of bilateral lower extremity weakness without a history of coagulopathy or anticoagulant use. The patient did fall around the time of symptom onset, possibly associating trauma with symptom presentation. The second case was further complicated by the patient's history of leukemia, leading to an initial suspicion of intradural malignancy.

Radiologically, hemorrhagic cysts often mimic epidural abscesses or neoplastic lesions because of T1 hyperintensity and postcontrast enhancement, which can delay diagnosis and surgical intervention.^6^ In our cases, imaging characteristics led to initial consideration of infectious or neoplastic etiologies, highlighting the limitations of imaging alone in differentiating hemorrhagic synovial cysts from other epidural pathologies.^1,4,7^

The pathogenesis of hemorrhagic transformation remains uncertain. Hypotheses include microvascular rupture because of minor trauma, chronic pressure changes, or anticoagulant-related bleeding. However, spontaneous hemorrhage without identifiable triggers has been documented, reinforcing the idea that vascular fragility within degenerative joints may predispose to this complication.^3,6,8^

In both cases, histopathological analysis was required for definitive diagnosis, confirming an organizing hematoma with no evidence of malignancy or infection. In published literature, histological findings commonly include synovial lining, hemosiderin deposits, neovascularization, and hemorrhage.^3,7,9^ These findings reinforce the importance of tissue analysis, particularly in cases with ambiguous imaging or acute symptoms.

Surgical resection remains the treatment of choice in symptomatic hemorrhagic cysts. Conservative management is generally ineffective once hemorrhage has occurred, especially with significant neurological deficit.^5,7,8^ Prompt decompression typically results in favorable outcomes, as seen in our cases where patients showed substantial postoperative recovery.

## CONCLUSION

Despite the limited sample size, our findings contribute to the evidence that hemorrhagic synovial cysts should be included in the differential diagnosis of acute spinal compression syndromes. The combination of multidisciplinary evaluation, strong radiological and histopathological correlation, and prompt surgical management is essential for achieving the favorable patient outcomes observed in our series.
